# Neurofilament light-chain (Nf-L) as a biomarker in seizures and status epilepticus of varying duration

**DOI:** 10.1007/s00415-026-13780-7

**Published:** 2026-04-02

**Authors:** Sophie Schlabitz, Jakob I. Doerrfuss, Jonas M. Hebel, Péter Körtvélyessy, Martin Holtkamp, Verena Gaus

**Affiliations:** 1 Department of Neurology, Charité - Universitätsmedizin Berlin, Corporate Member of Freie Universität Berlin, Humboldt-Universität Zu Berlin and Berlin Institute of Health, Berlin, Germany; 2 Department of Neurology, Charité - Universitätsmedizin Berlin, Center for ALS and Other Motoneuron Disorders, Corporate Member of Freie Universität Berlin, Humboldt-Universität Zu Berlin and Berlin Institute of Health, Berlin, Germany; 3 Epilepsy-Center Berlin-Brandenburg, Institute for Diagnostics of Epilepsy, Berlin, Germany

**Keywords:** Cerebrospinal fluid, Epilepsy, Neuronal damage, Serum

## Abstract

**Background:**

Neurofilament light chain (Nf-L) is an established biomarker for neuronal damage and a prognostic parameter in many diseases of the central nervous system (CNS). In epilepsy, most studies have focused on Nf-L levels either in epileptic seizures or in status epilepticus. We sought to investigate the correlation between epileptic seizures of different duration and elevated Nf-L levels as a biomarker for neuroaxonal injury also considering methodological confounders in Nf-L measurement.

**Methods:**

In adult patients, Nf-L was investigated in serum and/or cerebrospinal fluid (CSF) after a single epileptic seizure (51 patients), acute repetitive seizures (11 patients) or status epilepticus (20 patients) along with standard acute CSF parameters. In addition, ratios of Nf-L to age-adjusted reference intervals were calculated.

**Results:**

In both serum and CSF, absolute Nf-L levels were significantly increased after status epilepticus compared to patients who experienced a single seizure (serum: *p* < 0.0001; CSF: *p*  = 0.0008) or acute repetitive seizures (serum: *p* = 0.035 CSF: *p*  = 0.045). Similarly, age-adjusted Nf-L ratios in serum were higher after status epilepticus (median = 1.68, interquartile range = 2.81) than after a single seizure (median = 0.67, interquartile range = 0.73; *p*  = 0.039). Though ANOVA-testing indicated significant differences in CSF Nf-L ratios between the three groups (*p*  = 0.040), pairwise post hoc testing was not significant. A moderate significant positive correlation was observed between the duration of status epilepticus and serum Nf-L concentrations (Pearson r = 0.48, *p*  = 0.02).

**Conclusion:**

Our data indicate that single and acute repetitive seizures likely do not cause significant neuronal injury as assessed by Nf-L concentrations in serum and CSF. In contrast, status epilepticus is accompanied by a duration-dependent increase in serum Nf-L, which presumably reflects seizure-associated cellular injury and underlines the need for rapid and consistent antiseizure treatment.

## Introduction

Neurofilament light chain (Nf-L) is a subunit of the neurofilament protein. Neurofilaments are highly specific scaffolding proteins of neuronal axons in the central and peripheral nervous system. They provide axonal support, ensure the axonal diameter and thereby also have an indirect influence on nerve conduction velocity [1, 2]. Damage to the axonal cell membrane leads to the release of Nf-L into the interstitium and, consequently, to the cerebrospinal fluid (CSF) and blood/serum [3]. Serum Nf-L reference (cut-off) values increase with advancing age in adults [4, 5]. Additionally, Nf-L serum levels are affected, i.e., elevated, by renal impairment, as Nf-L elimination also depends on renal filtration [6].

In clinical routine, Nf-L in CSF or serum has become an established biomarker for diagnostic classification and prognostic assessment of several chronic neurological—especially neurodegenerative – diseases, such as amyotrophic lateral sclerosis (ALS), frontotemporal dementia (FTD) and multiple sclerosis [7, 8]. After acute damage of the central nervous system, like traumatic brain injury (TBI), increase of Nf-L levels in both CSF and serum is detectable within hours. Nf-L levels then further increase thoroughly. In TBI, they peak after 10 days in CSF and after 20–30 days in serum [9, 10].

In the diagnostic workup of epileptic seizures, established biomarkers, such as lactate and creatine kinase (CK), merely reflect muscular activity associated with the tonic phase of a seizure or convulsions but do not permit conclusions about the extent of neuronal excitation or the resulting neuronal damage. Moreover, their elevation is detectable in serum only for a relatively brief period of time of several hours (lactate) to a few days (CK) [11, 12]. Accordingly, laboratory-based retrospective diagnostic clarification is only possible within a limited time window. Prolactin, a hormone secreted predominantly by the pituitary gland, did not prove to be a suitable biomarker of seizures, mainly because of interindividual variability in normal ranges and intraindividual circadian fluctuations as well as a relevant rate of positive findings in syncope [13].

Nf-L may therefore represent a promising novel biomarker for the diagnostic classification of epileptic seizures, due to its rapid detectability with prolonged persistence for several months [14]. Furthermore, it could contribute to the pathophysiological understanding of the extent to which epileptic seizures may cause neuronal damage. Evidence on the diagnostic value of Nf-L in epilepsy is inconsistent and mostly based on serum measurements. Whereas single or self-limited seizures are generally not associated with sustained serum Nf-L elevation, higher levels have been reported in status epilepticus [15–22].

With this study, we aim to contribute further evidence supporting the applicability and diagnostic value of Nf-L as a biomarker for neuronal injury after seizures. We hypothesise that Nf-L concentrations differ according to seizure severity and would be highest after status epilepticus compared to single or acute repetitive seizures. Our primary endpoints were the serum and CSF Nf-L concentrations across these seizure entities. Special emphasis is placed on minimising common methodological confounders of Nf-L measurements within the cohort, particularly age and renal function. Our secondary outcome of interest was the potential alteration of standard variables in CSF (cell count, protein, glucose and lactate) in the three seizure entities.

## Methods

### Study design and setting

This study was conducted at the Department of Neurology at Charité – Universitätsmedizin Berlin, Germany. All data were primarily obtained for clinical purposes. Retrospective data extraction and analyses were approved by the ethics committee of Charité – Universitätsmedizin Berlin (Application No. EA2/263/23).

Using our hospital’s IT system, we reviewed medical charts and identified patients aged 18 years and older who were diagnosed with a single seizure, acute repetitive seizures or status epilepticus (SE) between the 1st of November 2020 and the 30th of April 2023. We considered SE according to the definition of the German Neurological Society guidelines as any seizure lasting more than 5 min regardless of its semiology [23]. As common in German clinical practice, we defined acute repetitive seizures as the occurrence of two or more seizures within 24 h with return to consciousness between seizures [24]. Based on the current seizure classification of the International League Against Epilepsy [25], a semiological distinction was made between seizures with preserved and impaired consciousness. Since the data were gathered during the validity of the previous seizure classification [26], we also categorised the seizures as motor and non-motor. Additionally, with respect to the potential impact of the amount of involved cortex on the Nf-L levels, we chose to also distinguish between regional seizures (i.e., focal seizures presumably limited to one hemisphere according to semiology) and bihemispheric seizures meaning involvement of both hemispheres at some stage of the seizure, regardless of focal or generalised seizure onset. Since all seizures assessed in our study were at least in parts convulsive, we were able to make a purely clinical diagnosis. For the purpose of this study, all seizure-related clinical information, including classification and semiology obtained during routine clinical care, were re-evaluated by an experienced epileptologist (SS).

Patients with acute neurological diseases within the last 6 months or with chronic neurodegenerative conditions were excluded from our analysis, as an increase in Nf-L concentration may be caused by the underlying disease, namely ischaemic or haemorrhagic strokes [27], brain tumor [28], TBI [14], cerebral autoimmune diseases [29], infectious meningoencephalitis [30], severe peripheral neuropathy [31] and ALS [8], multisystemic atrophy, FTD, corticobasal degeneration and progressive supranuclear palsy [7]. As impaired renal function is known to be associated with Nf-L accumulation, patients with renal insufficiency stage 4 and 5 (eGFR < 30 ml/min), were also excluded from this study [6].

### Clinical data

We collected the following clinical data from the medical records: age; sex; pre-existing diseases and medications; date, duration, number and semiology of seizures and latency from seizure onset to sampling. In addition, we incorporated the following paraclinical data as available: laboratory findings in serum and CSF; electroencephalogram and neuroimaging (MRI and CT).

### CSF parameters

When indicated within clinical routine, CSF specimen were drawn for diagnostic evaluation. The standard CSF parameters cell count, protein, glucose and lactate were determined routinely with established standard procedures: Electrical impedance for cell count, immunturbidimetry for protein and photometry for lactate and glucose measurement.

### Nf-L measurement

Nf-L levels were analysed in serum and CSF samples. For some patients, values from several time points up to a maximum of 1 month after seizure onset were available; in these cases, mean values were calculated for further analysis. Absolute Nf-L levels and ratios of Nf-L to age-adjusted reference intervals were determined in serum and CSF [32]. Both were logarithmically transformed to further improve the correlation with age.

Nf-L levels in serum were measured by a fully automated HD-X-Analyzer (Quanterix, USA) based on a single-molecule array (Simoa®) using a commercially available kit (NF-Light Advantage Kit V1-V3). Serum Nf-L values were normalised using the following ratio based on established age-partitioned reference ranges [32].

$$\begin{aligned}&{\mathrm{Nf}} - {\text{L ratio }}\left( {{\mathrm{Serum}}} \right) \\&= \frac{{{\text{individual Nf}} - {\text{L concentration}}}}{{97.5\%{\text{ percentiles of reference range }}\left( {4.19{\text{ x }}1.029{^{age}}} \right)}}\end{aligned}$$

An enzyme-linked immunosorbent assay (NF-Light™ CSF ELISA assay, UMAN Diagnostics, Sweden) was applied to quantify Nf-L concentrations in CSF, which were normalised using the following ratio and internal laboratory reference values used in clinical practice of our hospital (Table 1).

$${\mathrm{Nf}} - {\text{L ratio }}\left( {{\mathrm{CSF}}} \right){ } = \frac{{{\text{individual Nf}} - {\text{L concentration}}}}{{\text{upper limit of reference range}}}$$

**Table 1:** Age-specific CSF reference limits for Nf-L

Age (years)	Upper limit of reference range
< 30	$\le$ 289 pg/ml
30 to < 39	$\le$ 379 pg/ml
39 to < 60	$\le$ 829 pg/ml
≥ 60	$\le$ 2200 pg/ml

### Statistics

Raw data were analysed with MS Excel 16. Statistical analyses and processing of graphs were performed using GraphPad Prism 10 (GraphPad Software, La Jolla, CA, USA). Absolute Nf-L levels and age-adjusted ratios in serum and CSF were log-transformed for subsequent analyses (log 10 = lg). All data were first tested for normality distribution with D´Agostino and Pearson omnibus normality test. To assess statistical significance between groups, continuous variables were examined using ordinary one-way ANOVA with multiple comparisons (normally distributed data) or Kruskal–Wallis test (non-normally distributed data or small sample size) and post hoc Tukey´s or Dunnett´s multiple comparison of individual groups. Correlations between absolute Nf-L concentration in serum and CSF as well as Nf-L ratios and the duration of status epilepticus were tested for linear correlation using the Pearson correlation coefficient. Values of *p* < 0.05 were considered statistically significant. All data analysed by parametric tests are expressed as mean ± standard deviation while data analysed by non-parametric tests are shown as median with interquartile range. OmniGraffle 7 (The Omni Group, Seattle, WA, USA) was used as graphical software to process images.

## Results

### Clinical characteristics of patients

**Fig. 1:**
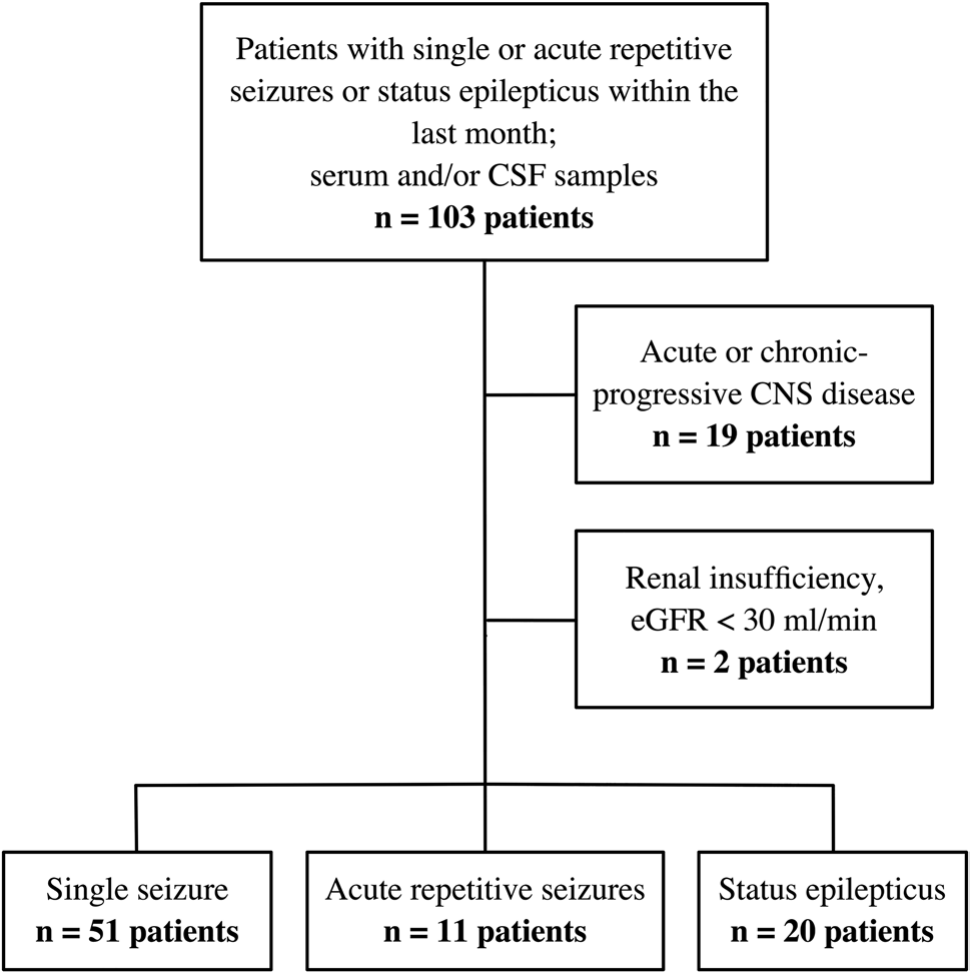
Flow chart of patient assessment. Of 103 admitted patients, 19 were excluded due to acute CNS disease within the last 6 months or a chronic progressive neurological disorder and 2 due to impaired renal function. Among the remaining 82 patients, 51 were diagnosed with a single seizure, 11 with acute repetitive seizures and 20 with status epilepticus. Abbreviations: CFS = cerebrospinal fluid; CNS = central nervous system; eGFR = estimated glomerular filtration rate

A total of 103 patients were eligible for this study, of which 19 were excluded due to an acute brain disease or chronic progressive neurological disorder and 2 due to severe chronic renal insufficiency (eGFR < 30 ml/min) (Fig. 1). Out of 82 patients, 51 had experienced a single seizure, 11 acute repetitive seizures and 20 SE. The characteristics of patients including clinical information on seizure semiology and aetiology are reported in Table 2.

**Table 2:** Overview of clinical characteristics, cerebrospinal fluid parameters and Nf-L levels in the different groups

	Single seizure N = 51	Acute repetitive seizures N = 11	Status epilepticus N = 20	Significance
**Sex, n (%)**				
Female	24 (47.1)	5 (45.5)	8 (40.0)	*p* = 0.867
Male	27 (52.9)	6 (54.5)	12 (60.0)	
**Age, years**				
Mean (± SD)	48 (± 18.0)	58 (± 19.1)	65 (± 17.4)	***p* = 0.014**
Range	18 – 82	29—84	25—91	
**Semiology, n (%)**				
Non-motor seizures	0 (0)	0 (0)	0 (0)	
Motor seizures	51 (100)	11 (100)	20 (0)	
Seizures with preserved consciousness	0 (0)	0 (0)	0 (0)	
Seizures with impaired consciousness	51 (100)	11 (100)	20 (0)	
**Cortical involvement, n (%)**				
Regional seizures	1 (2.0)	0 (0)	5 (25.0)	
Bihemispheric seizures	50 (48.0)	11 (100)	15 (75.0)	
**Aetiology, n (%)**				
Acute symptomatic seizure	0 (0)	2 (18.2)	0 (0)	
Isolated unprovoked seizure	22 (43.1)	1 (9.1)	2 (10.0)	
Seizure in context with epilepsy	29 (56.9)	8 (72.7)	18 (90.0)	
**Laboratory analyses, n (%)**				
Serum	49 (96.1)	9 (81.1)	17 (85.0)	
CSF	28 (54.9)	8 (72.7)	13 (65.0)	
**Latency of sampling, days**				
Serum, median (IQR)	1.1 (0.4 – 2.6)	1.3 (0.6 – 2.8)	2.8 (1.6 – 7.0)	***p* = 0.028**
CSF, median (IQR)	0.9 (0.3 – 2.1)	0.8 (0.3 – 1.1)	0.4 (0.2 – 3.4)	*p* = 0.505
**CSF parameters**				
Cell count (/µl), median (IQR)	1.5 (0.3—2.0)	1.5 (0.3—2.0)	2.0 (0.5—4.5)	*p* = 0.660
Protein (mg/l), mean (± SD)	372.6 (± 145.0)	454.2 (± 127.3)	513.9 (± 146.2)	***p* = 0.016**
Lactate (mg/dl), mean (± SD)	16.8 (± 3.5)	21.3 (± 5.0)	26.3 (± 9.7)	***p* < 0.0001**
Glucose (mg/dl), median (IQR)	68.0 (63.3—74.5)	73.0 (65.8—106.8)	89.0 (70.5—108.5)	***p* = 0.046**
**Nf-L absolute, pg/ml**				
Serum, median (IQR)	11.5 (7.1 – 21.6)	17.4 (10.9 – 33.3)	61.9 (13.6 – 114.0)	***p* = 0.003**
Serum, range	4.2 – 71.6	4.7 – 48.1	3.6 – 277.7	
CSF, median (IQR)	536 (358 – 1072)	705 (514 – 1320)	1552 (1043 – 5909)	***p* = 0.006**
CSF, range	116 – 5824	290—1615	259—27,218	
**Ratio of Nf-L to age-adjusted reference**				
Serum, median (IQR)	0.67 (0.51 – 1.25)	0.79 (0.62 – 1.01)	1.68 (0.61 – 3.41)	***p* = 0.045**
CSF, median (IQR)	0.62 (0.43 – 1.36)	0.61 (0.40 – 0.78)	1.23 (0.68 – 2.70)	***p* = 0.040**
**Participants exceeding**^**1**^** age-adjusted values, n (%)**				
Serum	15 (30.6)	2 (22.2)	10 (58.8)	*p* = 0.077
CSF	10 (37.0)	0 (0)	8 (61.5)	***p* = 0.035**

CSF = cerebrospinal fluid; IQR = interquartile range; SD = standard deviation

^1^Exceedance of age-adjusted reference values is defined as serum levels above the 97.5th percentile and CSF levels above age-defined cut-off values

### Nf-L in serum and CSF after seizures

**Fig. 2:**
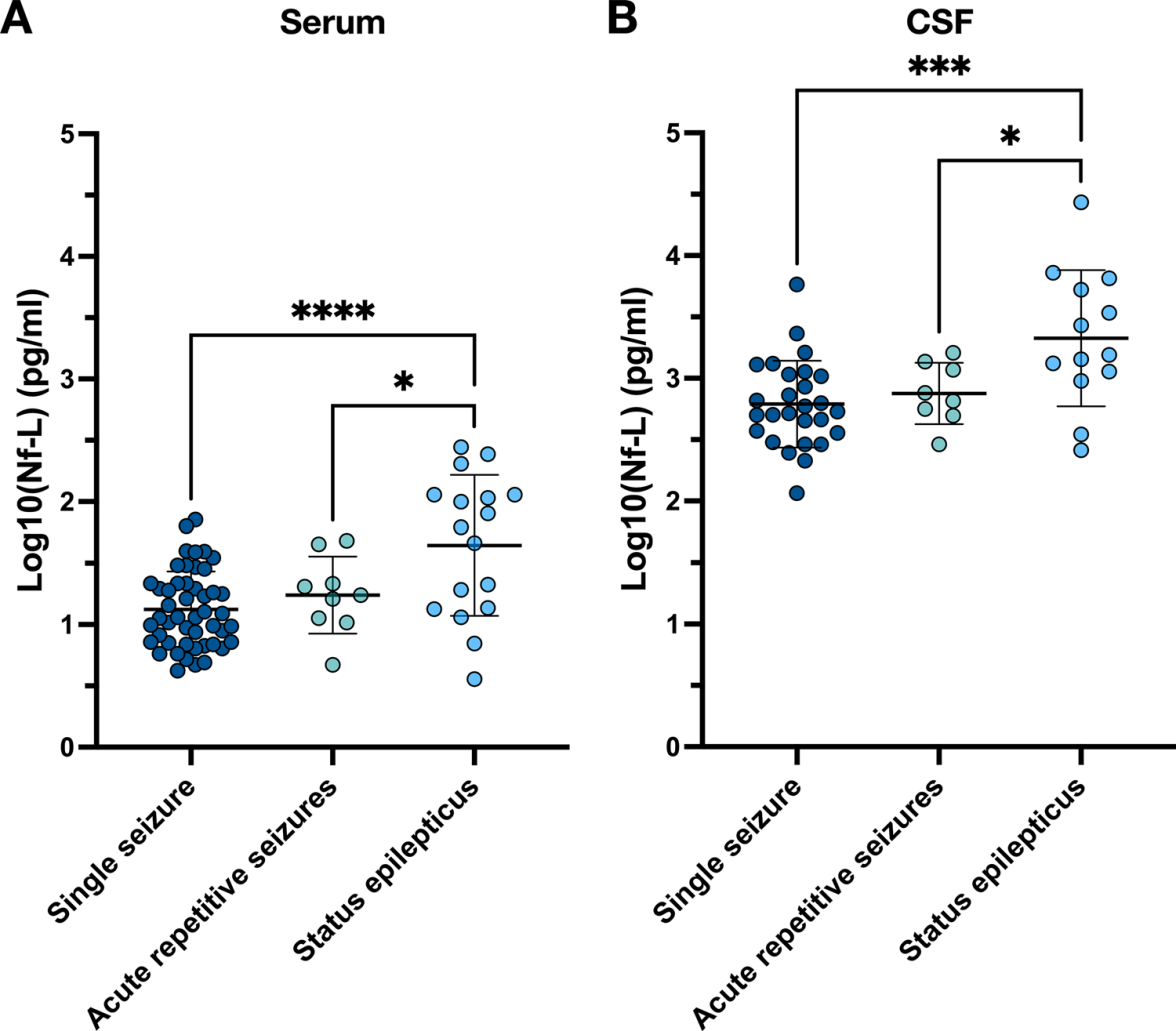
Absolute Nf-L concentrations (pg/ml) measured in (**A**) serum and (**B**) cerebrospinal fluid (CSF) in patients with a single seizure (serum: n = 49; CSF: n = 27), acute repetitive seizures (serum: n = 9; CSF: n = 8) or status epilepticus (serum: n = 17; CSF: n = 13). Nf-L levels are shown as log10-transformed values. Each dot represents an individual patient; dashes indicate mean ± standard deviation for each group (*p < 0.05, ****p < 0.0001)

**Fig. 3:**
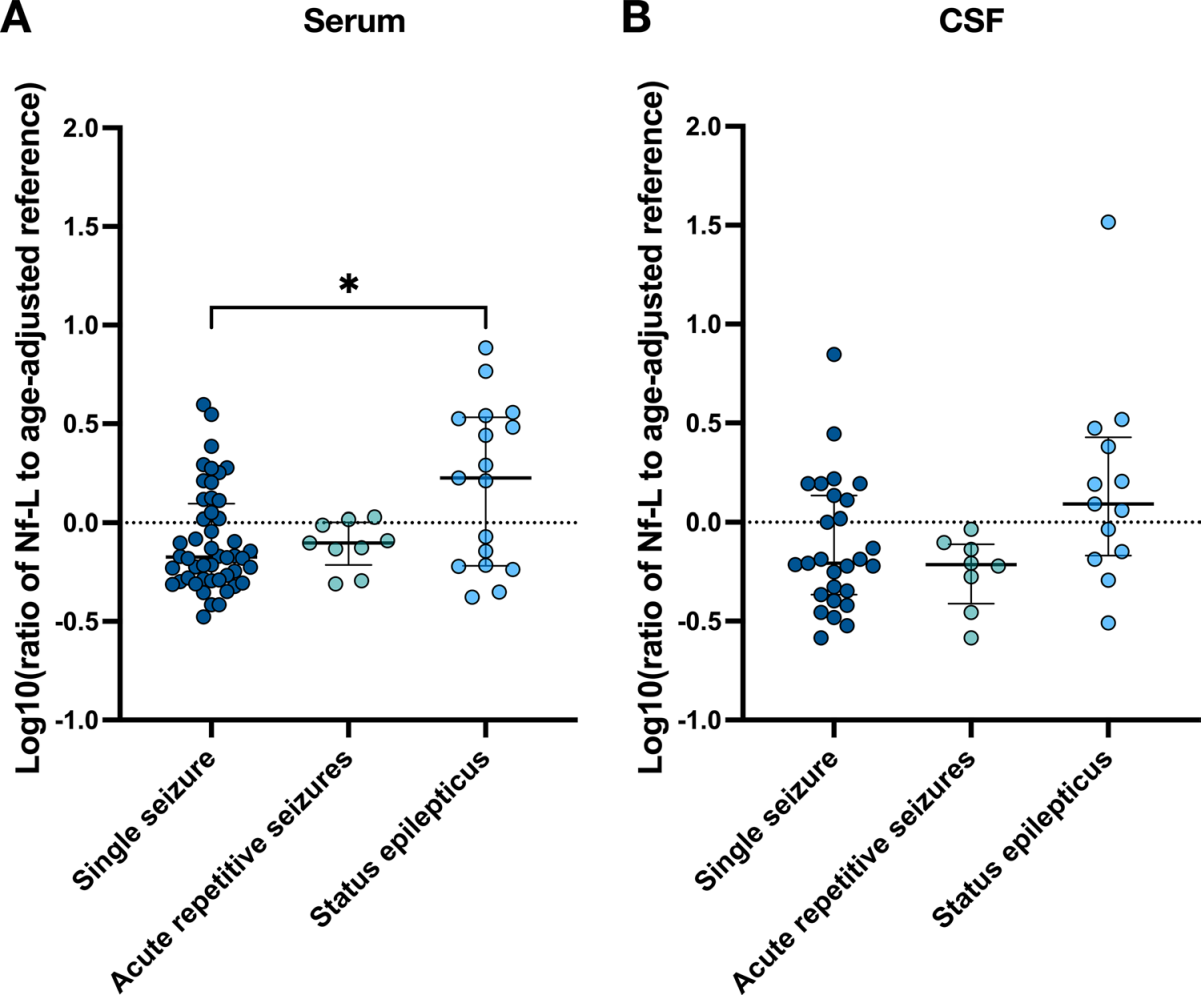
Ratios of individual Nf-L concentrations to age-adjusted reference intervals determined in (**A**) serum and (**B**) cerebrospinal fluid (CSF) in patients with a single seizure (serum: n = 49; CSF: n = 27), acute repetitive seizures (serum: n = 9; CSF: n = 8) or status epilepticus (serum: n = 17; CSF: n = 13). Ratios are shown as log10-transformed values. Each dot represents an individual patient; dashes indicate median (interquartile range) for each group (*p < 0.05)

We analysed Nf-L levels in 88 serum and 50 CSF samples of 82 patients. In serum, duplicate and triplicate Nf-L measurements were available for 12 and 1 patients, respectively; the shortest and longest intervals between blood draws were 8 and 168 h. In CSF, Nf-L was determined in duplicate in only one patient with an interval of 154 h between lumbar punctures. In patients with more than one sampling in either CSF or serum, mean values were calculated for further analysis. After SE, absolute Nf-L concentrations were significantly higher in both serum and CSF compared to patients who had a single seizure (in serum, *p* < 0.0001; in CSF, *p* = 0.0008) or acute repetitive seizures (in serum, *p* = 0.035; in CSF, *p* = 0.044; Fig. 2). Considering the age-dependency of Nf-L, ratios of Nf-L to age-adjusted reference intervals were calculated for individual patients. Consistent with the absolute values, the age-adjusted serum ratios measured after SE were increased compared to patients with single seizures (SE: median = 1.68, interquartile range = 2.81; seizure: median = 0.67, interquartile range = 0.73; *p* = 0.039). Though ANOVA-testing indicated significant differences in CSF Nf-L ratios between the three groups (*p* = 0.040), pairwise post hoc testing was not significant (Fig. 3).

### Determinants of Nf-L concentrations after seizures

**Fig. 4:**
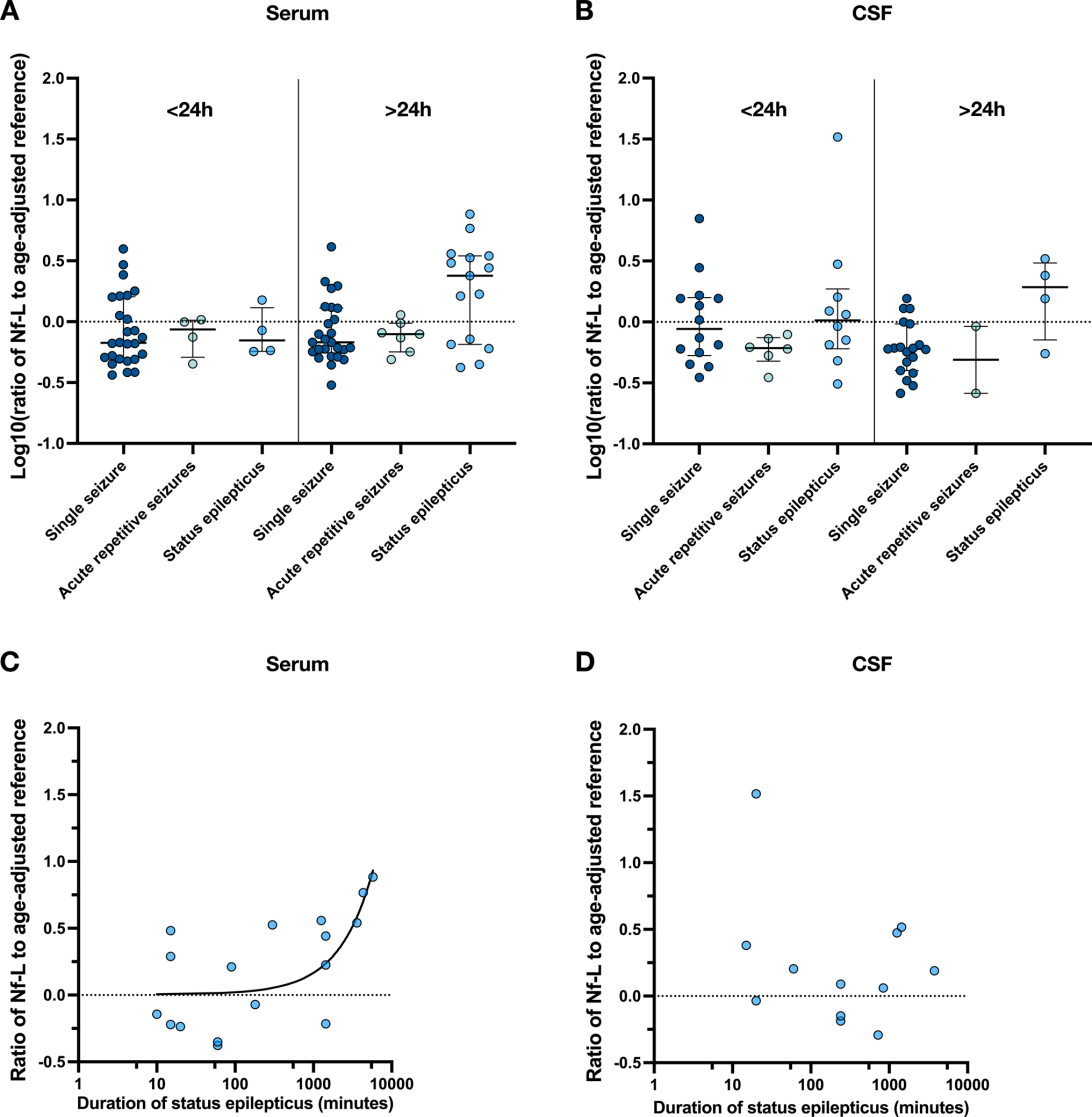
Ratios of individual Nf-L concentrations to age-adjusted reference intervals determined in (**A**) serum and (**B**) cerebrospinal fluid (CSF), stratified by the time interval between seizure onset and sampling (< 24 h, serum: single seizure n = 26, acute repetitive seizures n = 4, status epilepticus n = 4; CSF: single seizure n = 14, acute repetitive seizures n = 6, status epilepticus n = 10 versus > 24 h, serum: single seizure n = 20, acute repetitive seizures n = 7, status epilepticus n = 15; CSF: single seizure n = 13, acute repetitive seizures n = 2, status epilepticus n = 4). Nf-L ratios in (**C**) serum (n = 17) and (**D**) CSF (n = 12) in relation to the duration of status epilepticus. In serum, the regression line indicates a significant temporal correlation with a duration-dependent increase of Nf-L ratios (Pearson r = 0.48; *p* = 0.020). Ratios are shown as log10-transformed values. Each dot represents an individual patient; dashes in (A) and (B) indicate median (interquartile range) for each group

We found no correlation between the age-adjusted Nf-L ratios and the time elapsed between the estimated seizure onset and sample collection (Fig. 4). This time, the interval ranged in serum from 1.0 h to 17.5 days and in CSF from 2.0 h to 9.8 days.

When analysing Nf-L in relation to duration of SE, serum Nf-L ratios (n = 17) showed a significant time-dependent increase (Pearson r = 0.48; *p* = 0.020). At smaller group size (n = 12), this correlation was not seen in CSF (Fig. 4).

**Fig. 5:**
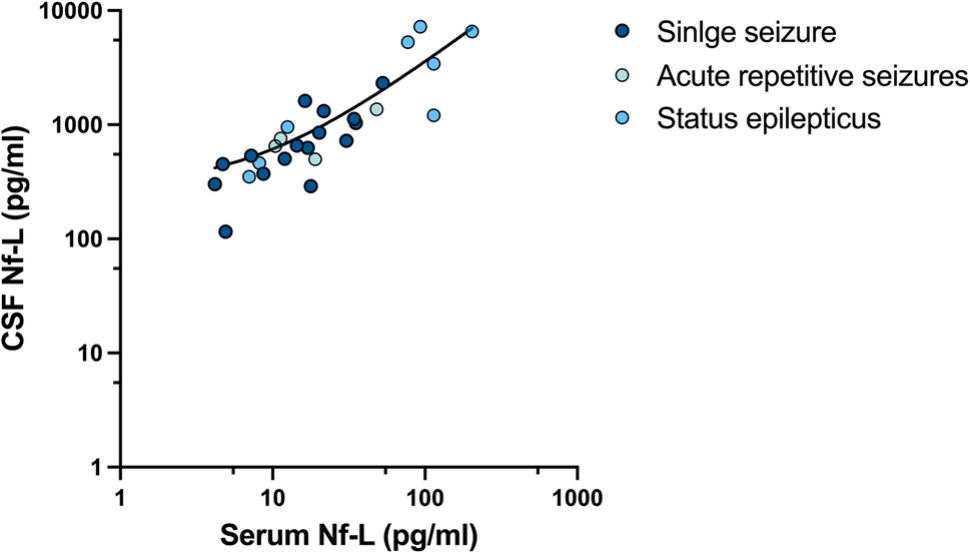
Correlation of Nf-L levels between simultaneously collected cerebrospinal fluid (CSF) and serum samples in patients with a single seizure (n = 16), acute repetitive seizures (n = 4) or status epilepticus (n = 8). No statistically significant group differences were observed (p = 0.847). Mean CSF/serum ratio was 48.8 ± 23.5, with a strong positive correlation (Pearson r = 0.81; *p* < 0.0001); values are shown on a log scale

Simultaneously collected serum and CSF samples were available in 28 patients, drawn between 2.0 h and 9.8 days after seizure onset. In this subgroup, there was a high correlation between absolute CSF and serum Nf-L levels with a mean ratio (CSF/serum) of 48.8 ± 23.5 (Pearson r = 0.81; *p* < 0.0001; Fig. 5).

Considering the presumed amount of cortical involvement, age-adjusted Nf-L ratios did not differ significantly between patients with regional and bihemispheric SE (in serum, *p* = 0.19; in CSF, *p* = 0.48).

### Standard CSF parameter findings in patients after seizures

**Fig. 6:**
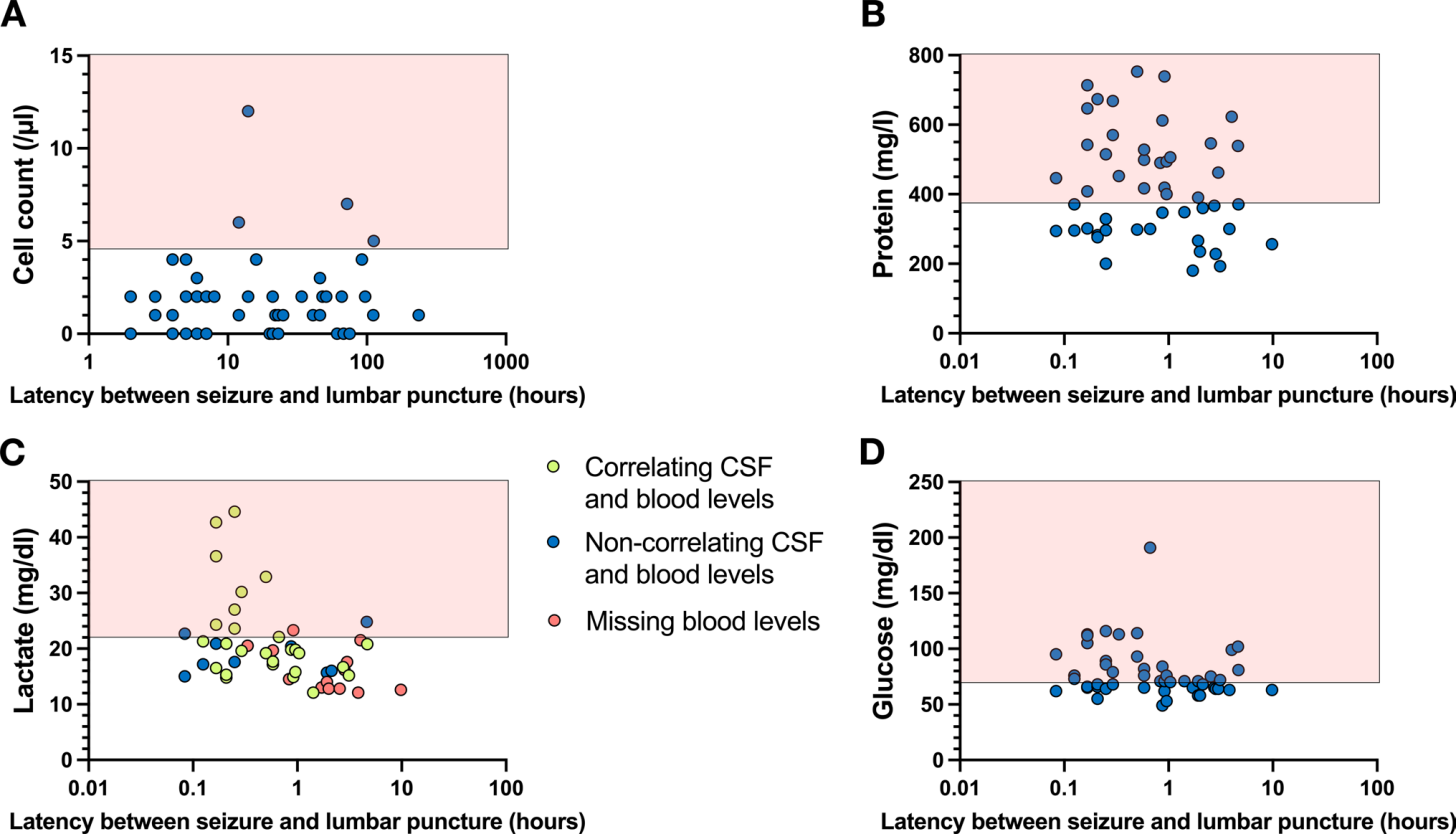
Results of cerebrospinal fluid (CSF) analysis in 49 patients who underwent lumbar puncture. CSF parameters including (**A**) cell count, (**B**) protein, (**C**) lactate and (**D**) glucose are presented in relation to the latency of lumbar puncture since seizure onset. Data points highlighted in red boxes indicate pathologically elevated values

A lumbar puncture was undertaken in 49 patients, the CSF results are shown in Fig. 6. The CSF cell count was elevated in 4 patients (8%) of our cohort, the maximal cell count was 12/µl (normal value < 5/µl). The cell count was not associated with the duration of seizure or SE. The lactate level was increased in 11 patients (22%), the highest lactate concentration was 44.6 mg/dl (normal levels < 22.0 mg/dl). Lactate values were dependent on the time interval between seizure manifestation and lumbar puncture. Blood lactate values were missing in 12 patients, in 27 of the remaining 37 patients (73%) a positive correlation between lactate concentration in CSF and the blood levels measured by arterial or venous blood gas analysis was found. The highest blood lactate concentration was 116 mg/dl (normal levels < 20.0 mg/dl). In 7 patients, elevated lactate levels in serum were not accompanied by an increase of lactate in CSF, with the shortest delay in lumbar puncture being 0.5 h and the longest 50 h. The glucose level (normal level < 70 mg/dl) was pathologically increased in 26 patients (53%) regardless of blood glucose. The protein value was elevated in 20 patients (41%), the maximal value was 753 mg/l (normal levels < 450 mg/l).

Lactate, glucose and protein values, but not the cell count, were significantly higher in patients with SE compared to those with single seizures (Table 2). In summary, the current data indicate that the CSF parameters lactate, glucose and protein levels are altered in a considerable percentage of patients with seizures whereas cell counts remain largely unaffected.

## Discussion

In this retrospective cross-sectional analysis, we investigated Nf-L concentrations in serum and CSF following single epileptic seizures, acute repetitive seizures and SE, considering age-adjusted reference values of Nf-L and renal function. Besides, we considered standard CSF parameters to contribute further evidence concerning (post-)ictal alterations in CSF and exclude confounders, i.e., acute CNS infections.

To our knowledge, this is the only study systematically comparing age-adjusted serum and CSF Nf-L levels across different seizure entities with varying duration.

We observed a significant increase in absolute Nf-L levels in both serum and CSF during SE compared to single seizures and acute repetitive seizures among participants with sufficient renal function (and unimpaired Nf-L clearance). These findings are consistent with previous retrospective studies that examined Nf-L in serum [21] and CSF [20, 22] after SE, in comparison to healthy controls and PWE in the interictal state. In contrast, another study reported a subtle but significant increase in serum Nf-L following single epileptic tonic–clonic seizures, with samples taken 2 and 6 h after presumed seizure onset [15], which was not observed in our cohort. Possible reasons for these differing results include the relatively small sample sizes in both studies (n = 20 in the previous study and n = 51 in our study), differences in age distribution between the studies, and the use of standardised sampling times in the other study. However, regarding the latter, neither previous studies nor our cohort found an association between the latency from seizure onset to sample collection [20], at least within the studied time frame.

To our knowledge, previous studies investigating Nf-L levels and their dynamics following epileptic seizures have addressed the issue of age dependence in Nf-L reference values primarily by employing age-matched controls [20] or by selecting study and control groups with a narrow, homogeneous age range [15]. In order to account for the significant influence of age and due to the markedly heterogeneous age distribution among our three groups — single seizures, acute repetitive seizures and SE — we calculated ratios of Nf-L to age-adjusted reference intervals for each patient. The age-adjusted serum Nf-L ratios after SE were consistently elevated compared to those after single seizures, mirroring the pattern observed in absolute values. In CSF, however, only a trend toward higher values after SE was observed, which may be explained by the significantly higher age in the SE group and the smaller sample size. Equal CSF and serum Nf-L ratios across the three diagnostic groups indicate that higher Nf-L values in SE are not caused by additional peripheral neuropathy, neither premorbid nor acquired during SE. These findings underscore the importance of adequate age correction of Nf-L values for accurate interpretation at the individual patient level.

As indicated above, similar to previous studies, we found no association between age-adjusted Nf-L ratios and the latency between estimated seizure onset and sample collection, with the earliest sampling time after 1 h (serum) and after 2 h (CSF). This suggests an early increase and detectability of Nf-L in serum following significant neuronal injury. Furthermore, the strong correlation between absolute Nf-L values in serum and CSF during simultaneous sampling starting from 2 h after onset indicates a rapid transfer of Nf-L released in the CNS into the serum, likely reflecting relevant blood–brain barrier disruption. These findings support the practical applicability of age-adjusted serum Nf-L ratios as a biomarker after epileptic seizures, without the additional need for measurement of Nf-L in CSF.

In our cohort as well, the duration of SE had a significant impact on Nf-L levels. We concur with previous authors who interpret this as evidence of time-dependent injury to the neuronal cytoskeleton caused by disinhibited excitation. In contrast, excitation during self-limiting epileptic seizures appears to have little measurable effect on scaffolding neuronal proteins. However, it has been demonstrated that glial injury, as indicated by an increase in glial fibrillary acidic protein (GFAP), can be detected even after self-limiting epileptic seizures [15]. We do not share the hypothesis that the duration of hypoxia during seizures is primarily responsible for the extent of Nf-L elevation, since in convulsive SE, hypoxia is typically addressed early through airway management and ventilation, even if ictal excitation remains refractory to therapy. Nevertheless, we still observed a correlation between increasing SE duration and Nf-L levels. Furthermore, a study investigating Nf-L after severe cerebral hypoxia in the context of hypoxic-ischaemic encephalopathy following cardiac arrest showed that the presence of additional electrographic SE (ESE) significantly increased the already elevated Nf-L levels [33]. However, it should be noted, that the criteria for diagnosing ESE in this study were broader than those recommended in epilepsy guidelines [23, 34, 35].

Interestingly, the extent of presumed neuronal involvement in ictal activity, which we classified conceptually based on regional versus bihemispheric ictal patterns, had no impact on Nf-L levels. This finding may be influenced by the small sample size as well as by clinically inapparent ictal propagation.

Regarding standard CSF parameters, our analysis supports, on the one hand, the finding that CSF pleocytosis following seizures is rare: in our cohort, elevated cell counts were observed in 8% of patients, consistent with other studies reporting an incidence of 3 and 10% [36–39]. The cell count increase was small, likely indicating an unspecific co-phenomenon of the seizures.

On the other hand, metabolic parameters including CSF lactate, glucose, and protein levels were altered in a considerable proportion of our patients—particularly those with SE. The observed increase in CSF lactate after seizures and its correlation with serum lactate aligns with previous studies [37, 40]. As described before, our data also indicate that CSF lactate levels peak early after a seizure and decline with time [37]. Our results further confirm existing data of significantly higher CSF protein values in patients with SE compared to single seizures [36–38, 41]. Unlike prior studies, which rarely noted pathological CSF glucose abnormalities (≈6%) and more often reported a decreased CSF/serum glucose ratio after SE, we observed significantly elevated CSF glucose levels in patients with SE [40, 42]. The interpretation of this finding, however, is limited by the absence of CSF/serum glucose ratio measurements in our cohort, which restricts direct comparability with earlier work and constrains the pathophysiological interpretation. A plausible contributing factor to the higher CSF glucose values in our SE group may be the older age of these patients compared to those experiencing single seizures, as age-related metabolic differences could influence CSF glucose dynamics.

Overall, our findings support the hypothesis that postictal CSF changes are metabolic or barrier-mediated rather than inflammatory and must be interpreted in relation to the duration and timing of the seizure.

When interpreting our results, the limitations of our study have to be kept in mind: First, the retrospective single-center design inherently introduces information and selection bias hampering generalisability. Second, the timing of serum and CSF sampling varied widely, ranging from hours to several days after seizure onset and limits the interpretation of temporal associations. CSF analyses were available for only a subset of patients. Third, group differences in age—especially the higher age in patients with SE—may have influenced biomarker levels despite age-adjusted normalisation. Finally, the relatively small sample sizes in some subgroups reduce statistical power, particularly for CSF-based analyses.

## Conclusions

In this retrospective study, we found no hints for significant neuroaxonal injury, as reflected by elevated serum Nf-L levels, in patients with self-limiting single seizures or acute repetitive seizures. In contrast, status epilepticus was associated with a duration-dependent increase in serum Nf-L, likely reflecting cellular injury and molecular alterations induced by prolonged neuronal excitation, thereby underscoring the need for rapid and consistent treatment of this neurological emergency. Overall, Nf-L appears to be a promising biomarker of neuroaxonal injury in prolonged seizures; however, age- and renal function–dependent metabolism, as well as potential confounding by concomitant CNS pathology or peripheral neuropathy, must be considered when interpreting Nf-L levels. The predictive values of Nf-L elevation in SE for functional outcome should be investigated in future prospective longitudinal studies.

## Data Availability

Anonymized data will be made available upon request to any qualified investigator.
